# Comparison of Glycosylated Haemoglobin, Blood Pressure, and Anthropometric Measurements Depending on Gender and Bodyweight State in Adolescents

**DOI:** 10.3390/children9121922

**Published:** 2022-12-08

**Authors:** Jorge Carlos-Vivas, Antonio Castillo-Paredes, Rafael Gómez-Galán, Laura Muñoz-Bermejo, María Mendoza-Muñoz, Jose Carmelo Adsuar, Raquel Pastor-Cisneros, Violeta Calle-Guisado

**Affiliations:** 1 Promoting a Healthy Society Research Group (PHeSO), Faculty of Sport Sciences, University of Extremadura, 10003 Cáceres, Spain; 2 Grupo, Investigación en Actividad Física y Salud Escolar, Escuela de Pedagogía en Educación Física (AFySE), Facultad de Educación, Universidad de Las Américas, Santiago 8370040, Chile; 3 Research Group on Physical and Health Literacy and Health-Related Quality of Life (PHYQOL), University of Extremadura, 06800 Badajoz, Spain; 4 Social Impact and Innovation in Health (InHEALTH) Research Group, Faculty of Sport Sciences, University of Extremadura, 10003 Cáceres, Spain; 5 Departamento de Desporto e Saúde, Escola de Saúde e Desenvolvimento Humano, Universidade de Évora, 7004-516 Évora, Portugal

**Keywords:** adolescents, anthropometry, bodyweight, blood pressure, glycosilated haemoglobin, health

## Abstract

Background/Objective: The greatest anthropometric and physiological changes occur during adolescence. Assessment of growth patterns is necessary to prevent future health risks. Aims: To describe the values of glycosylated haemoglobin (HbA1c), systolic (SBP) and diastolic (DBP) blood pressure, triceps skinfold, and abdominal circumference according to gender and age (between 12 and 17 years), as well as explore the differences between body weight conditions. Methods: A descriptive cross-sectional study was carried out, including 4130 adolescents between 12 and 17 years old. SBP and DBP, HbA1c, triceps skinfold, and abdominal circumference were evaluated. Results: Significant differences were observed between males and females for HbA1c (*p* < 0.001), SBP (*p* < 0.001), triceps curl (*p* < 0.001), and abdominal circumference (*p* < 0.001), independently of the age group. Regardless of gender and age groups, significant differences were observed between overweight/obese and normal-weight adolescents in SBP (*p* < 0.001), DBP (*p* < 0.001 to 0.009), triceps skinfold (*p* < 0.001), and abdominal perimeter (*p* < 0.001). Conclusions: Our findings revealed higher SBP, DBP, triceps skinfold, and abdominal circumference in overweight/obese adolescents compared to normal-weight adolescents in both genders.

## 1. Introduction

Cardiovascular disease (CVD) is the leading cause of death worldwide. CVD risk factors include high cholesterol, blood glucose levels, and obesity [1]. As blood pressure is considered an important indicator of cardiovascular health status, elevated blood pressure levels will be an important risk factor for public health [2]. Considering the Global Burden of Diseases, Injuries and Risk Factors (GBD) Study 2019 [3], high systolic blood pressure (SBP) leads to the 87 risk factors listed that account for an estimated 10.8 million deaths.

In recent years, the prevalence of hypertension has increased markedly in adolescents. At this stage, elevated blood pressure levels are often influenced by weight or genetic factors [4]. Obesity is a key determinant of elevated BP in children and adolescents [5,6,7]. The association between adiposity and disease risk begins early in life. Measures of obesity and adiposity, blood pressure, and other markers of macro- and micro-vascular function during childhood have been related to subsequent cardiovascular morbidity and mortality [8,9]. In addition, adipose tissue stores have different metabolic activities and relationships to disease risk depending on their distribution in the body [10]. Within the metabolic complications and adverse health effects from intra-abdominal adipose tissue, it can be found high blood pressure, hyperinsulinaemia, type 2 diabetes, and dyslipidaemia [11].

Anthropometric has been widely used to screen for cardiometabolic risk in children and adolescents, mainly due to its low cost, ease of administration, and non-invasive nature [12]. Furthermore, an estimated five million deaths are attributed to body mass index (BMI) values over 20–25 kg/m^2^.

Although BMI could be the measure used to define levels of overweight and obesity, it should be considered an indicator of weight but not adiposity [13] as it does not provide information on regional fat distribution and body proportions [14,15]. Furthermore, the BMI can be altered by variations in body water content, bone mass or muscle mass, which can lead to misclassifications, especially in children and adolescents with high muscle development [16,17]. In this sense, the body fat excess is the parameter that is mainly related to the risk of chronic diseases instead of the presence of overweight [18].

Waist circumference is a measure of central or visceral adiposity that may be a good indicator of adiposity, as well as a better predictor of elevated BP and risk of suffering cardiovascular diseases (CVD) in children than BMI [10,19,20,21,22]. However, waist circumference measurement may be limited in adolescents due to growth and changes in body composition [21]. Moreover, a relationship between waist circumference and skinfolds with increased blood pressure has been observed in children [23]. Therefore, supplementing waist circumference with hip circumference, waist-to-hip ratio, neck circumference, and skinfolds could be used to predict cardiovascular risk [24,25].

The greatest anthropometric, hormonal, and psychological changes occur between 12 and 18 years old, coinciding with adolescence. At this stage, being overweight was linked with a superior prevalence of high-level diastolic blood pressure (DBP), cholesterol, triglycerides, and glycosylated haemoglobin (HbA1c) in fasting blood [26,27]. For the treatment of obesity, they stress the importance of recognising excess body weight and central adiposity as health risks [23].

Therefore, knowledge of anthropometric development in pre-adolescent and adolescent stages is essential to prevent the appearance of health risks. Thus, this study aims to examine the values of HbA1c, DBP, SBP, triceps skinfold, and abdominal circumference according to gender and age in adolescents between 12 and 17 years old, as well as explore the possible differences between different body weight conditions (normal-weight vs overweight/obese).

## 2. Materials and Methods

### 2.1. Design

A descriptive cross-sectional study was conducted during a 14-month period from October 2007 to December 2008. A multistage stratified sample was utilised. The units of the successive stages were districts, health areas, educational centres (IESO), city typology (urban/rural), academic year, gender, and students (surveyable). The sample was selected from forty secondary schools.

### 2.2. Participants

Forty secondary schools from the Extremadura region (Spain) take part in this study. Schools were randomly selected to ensure the selection of a representative sample from all health areas in Extremadura. Thus, 4130 teenagers (2128 boys and 2002 girls) between 12 and 17 years old [14.25 (1.48)] were evaluated. Individuals had to meet the following eligibility criteria: (1) age between 12 and 17 years old; (2) live in the Autonomous Community of Extremadura; (3) be authorised by their parents or legal guardians; and (4) to be agreeing to join in the study.

### 2.3. Ethics Approval

The procedures of the present study were approved by the Bioethics and Biosafety Committee at the University of Extremadura, in agreement with the Declaration of Helsinki guidelines (reference code 11/2006). Both participants and their respective parents or legal guardians signed an informed consent form accepting their participation in the study.

### 2.4. Sample Size

The sample size was estimated based on data from the Spanish National Statistics Institute (www.ine.es, consulted on 16 June 2021) [24]. The population in Extremadura for this age group was 74,239 (51.3% male, 48.7% female). Thus, to provide an estimation with a certain degree of reliability for the survey at the regional level, a sample of 4130 individuals aged between 12 and 17 was selected.

Sections are made within each stratum with probability proportional to stratum size. The proportional allocation between the strata of health areas was employed. Within each stratum, the size of each substratum (rural or urban area) was applied. Thus, taking the 74,239 individuals and assuming a 99.9% certainty, a 2% precision, and a 50% expected proportion, a total of 3928 participants would be sufficient to carry out this study. Computations were made through the equation:

$$n=\frac{N+Z_{\propto }^{2}\times p\times q}{d^{2}\times \left(N-1\right)+Z_{\propto }^{2}\times p\times q}$$

where *N* is the total population; $Z_{\propto }^{2}$ is equal to 3.29 (if 99.9% certainty); *p* is the expected proportion (5%; i.e., 0.05); *q* is 1 minus p (1–0.05; i.e., 0.05); and *d* is the precision (2%).

Particularly, this study involved a sample of 4130 individuals in this age range to obtain reliable survey estimates at the regional level. Each stratum was separated into sections with probability proportional to their size. Proportional allocation between health area strata was used. Thus, within each stratum, the size of the rural or urban area (sub-stratum) was applied. Individuals were randomly selected within the school from the target populations with proportional sampling in age and gender.

### 2.5. Procedures and Measures

Data collection was performed in schools by qualified and standardised healthcare professionals. Assessments were carried out under standardised conditions and according to the protocol collected in the Data Collection Procedure Manual, which was specifically developed for the Childhood Obesity Surveillance Initiative (COSI) [25]. To measure height and body weight, participants removed their shoes and socks and any heavy clothing or accessories. Height was determined with a stadiometer (Tanita Tantois, Tanita Corporation, Tokyo, Japan), which was placed on a vertical surface with the measures scale perpendicular to the ground. It was evaluated in a standing position, with shoulders balanced and arms relaxed along the body. The result was taken in centimetres, with an accuracy of one millimetre. Body weight was assessed with a bioimpedance meter (Tanita MC-780 MA, Tanita Corporation, Tokyo, Japan), recorded in kg to the nearest 100 g. BMI was computed through the equation: body weight (kg) divided by height squared (m^2^). Each participant underwent a single assessment of height and body weight.

The dependent variables were systolic BP (SBP) and diastolic BP (DBP), expressed in mmHg for age, gender, and height, in addition to the diagnosis of normotension and prehypertension [26].

The independent variables were metabolic control, determined by the HbA1c value, and weight category, measured by BMI [27].

Values ≤ 7.5% [28,29] were considered as adequate glycaemic control and >7.5% as poor glycaemic control for statistical analysis.

BMI was used as a measure of weight category, taking as reference the World Health Organization (WHO). Thus, it was classified as: low (≤1 standard deviation [SD]); normal (=1 SD); overweight (>1 SD to ≤2 SD); and obese (>2 SD) [30].

Blood pressure was assessed in a sitting position with a manual sphygmomanometer and a stethoscope. It was measured at three different times: at the beginning, in the middle and at the end of the interview. The mean of the second and third records was considered to define high blood pressure (HBP). Individuals with SBP > 140 and/or DBP > 90 mmHg were classified as hypertensive according to the 2018 European Society of Cardiology (ESC)/European Society of Hypertension (ESH) guidelines [31]. In addition, participants were considered hypertensive regardless of their blood pressure if they were on antihypertensives or undergoing lifestyle modification to control HBP.

SBP and DBP were assessed with an oscillometric device (Microlife 3AC1-1, Widnau, Switzerland) following the Fourth Report on the Diagnosis, Evaluation and Treatment of High Blood Pressure in Children and Adolescents recommendations [32]. The cuff size was adapted to the arm of each participant based on the manufacturer’s suggestions. All blood pressure measurements were evaluated in triplicate (separated by 1 min) in a quiet environment and with an appropriate cuff size, with the mean value used for analysis. During BP measurements, participants sat quietly in a room temperature-controlled environment. Participants were classified as prehypertensive (systolic > 120/diastolic > 80; above 90/140 hypertension, thus between 80 and 90/120 and 140) or normotensive (<120/80 mmHg) according to the Fourth Report on the Diagnosis, Evaluation and Treatment of High Blood Pressure in Children and Adolescents [32]. It is now suggested that adults, children, and adolescents with blood pressure levels equal to or greater than 120/80 mmHg should be considered prehypertensive.

The procedure was explained to each participant, and they were asked not to speak during the measurement. The patients remained seated, with their backs supported and feet placed on a flat surface, in a comfortable environment in a resting position for five minutes. The arm was uncovered up to the shoulder and rested on a horizontal surface at the heart level. Beforehand, the circumference of the arm was measured to meet the requirement of using the most appropriate cuff, with a width of 40% and a length of 80–100%. The arm cuff was centred on the brachial artery without over-tightening or leaving it too loose. The lower edge of the cuff was placed 3 cm above the antecubital fossa.

Subsequently, the maximum inflation level was determined using the Osler manoeuvre to avoid pain or discomfort during the measurement. The stethoscope was located over the brachial artery with the entire surface in contact with the skin, and then the arm cuff was inflated rapidly and steadily to the predetermined level, releasing air from the chamber at a rate of 2 mmHg per beat. SBP was determined when two continuous beats appeared (Korotkoff phase I) and DBP at the time when the sounds disappeared (Korotkoff phase V). An additional 10 mmHg was examined after the last beat to confirm the disappearance of the beats.

The waist circumference [33] was determined with an anthropometric tape posted horizontally, halfway between the costal margin and the iliac crest, with subjects standing. The reading was taken just after a smooth exhalation.

HbA1c was quantified by high-performance liquid chromatography [34] (Bio-Rad laboratories, Hercules, CA, USA).

The skinfold [33] thickness at the level of the triceps brachii was measured at the middle of the arm with the arm relaxed and hanging laterally with the shoulder joint in slight external rotation and the elbow extended. The crease formed parallel to the longitudinal axis, with the thumb and index finger of the left hand separated from the underlying muscle and measured at that point, placing the plicometer perpendicular to the crease.

### 2.6. Statistical Analysis

Statistical analyses were ran through SPSS (version 25.0; IBM SPSS Inc., Armonk, IL, USA). Descriptive statistics were executed for all parameters. Data are presented as means and standard deviation (SD).

Kolmogorov–Smirnov and Levene’s tests were applied for analysing the normality and homogeneity of data, respectively. After that, the Mann--Whitney U test was employed to analyse between-gender and between-bodyweight condition comparisons in all dependent variables due to the non-parametric distribution of data previously confirmed. The alpha level was fixed at *p* ≤ 0.05. Additionally, Hedge’s g effect size with a 95% confidence interval was also estimated to compute the magnitude of comparisons. Effect size thresholds were the following [24]: >0.2, small; >0.5, moderate; >0.8, large.

## 3. Results

Table 1 displays the statistical descriptive and between-gender comparison for HbA1c, SBP, DBP, triceps skinfold, and abdominal circumference considering the total sample. Overall, results showed significant differences between males and females were observed for HbA1c (*p* < 0.001), SBP (*p* < 0.001), triceps skinfold (*p* < 0.001), and abdominal perimeter (*p* < 0.001), independently of the age group. There were also meaningful differences in DBP between gender in the 15–17 age group. However, no differences were detected for DBP in the 12 to 14 years old group (*p* = 0.272).

Table 2 and Table 3 show the statistical descriptive and comparison between bodyweight conditions in males and females, respectively, for HbA1c, SBP, DBP, triceps skinfold, and abdominal circumference stratified by age group. Independently of gender and age group, significant differences were found between overweight/obese and normal-weight adolescents in SBP (*p* < 0.001), DBP (*p:* <0.001 to 0.009), triceps skinfold (*p* < 0.001), and abdominal circumference (*p* < 0.001). In contrast, no differences were observed between bodyweight conditions for HbA1c in males (*p* = 0.397 to 0.459) nor females (*p* = 0.429 to 0.563), independently of the age group.

## 4. Discussion

The main findings show significant differences in SBP, DBP, triceps skinfold, and abdominal circumference between overweight/obese and normal-weight adolescents. However, no differences are observed for glycosylated haemoglobin levels and DBP in children aged 15–17 years. Thus, overweight/obese children have higher SBP and DBP levels (except for those aged 15–17 years), a higher triceps skinfold, and a larger abdominal circumference. Similarly, previous investigations have also shown an association between anthropometrics and blood pressure in childhood [30,31]. Hypertensive children present greater values of body weight, BMI, abdominal and hip circumferences, fat mass, and fat-free mass in comparison with their normotension counterparts. This supports the direct relationship between obesity and hypertension in these people [32].

These findings are clinically relevant, as obesity and hypertension are associated with high-risk cardiovascular disease, increasing related morbidity and mortality in adulthood [30,35]. High blood pressure values in childhood may predict metabolic and structural changes at an earlier age. There is evidence that children with elevated blood pressure have a sustained risk of becoming hypertensive adults due to permanent damage to target organs [36,37,38].

Estimating the prevalence of abdominal or central obesity is becoming increasingly common. In fact, several studies have shown that children with excess abdominal fat are also at increased risk of having a more atherogenic lipid profile, higher blood pressure, greater carotid intima-media thickness, and even metabolic syndrome [39,40]. Therefore, the truncal pattern of subcutaneous fat distribution may be linked with obesity and high-level blood pressure in youth [41].

Triceps skinfold thickness was also significantly related to obesity/overweight and normal weight in children, independent of age and gender. Skinfold thickness measurements represent an indirect measure of subcutaneous adipose tissue and are used to estimate total body density to derive body fat percentage [42]. Increases in BMI, body fat percentage, and skinfold thickness were related in an age range of 7 to 18 years, although they occurred in age- and gender-specific patterns [43]. Furthermore, the relationship between subscapular skinfold thickness and triceps skinfold thickness was associated with elevated blood pressure [41,44].

Previous findings [45] suggest that young people experience a mild elevation of HbA1c in adolescence. This elevation in HbA1c may be related to pubertal changes in the adipose tissue metabolism [46]. In this study, 7.5% has been taken as the acceptable limit as a protective factor for disease prevention in adulthood [47]. In this regard, no differences were found between HbA1c levels in overweight/obese boys and normal-weight boys.

On the other hand, due to boys having higher values for SBP, abdominal circumference, and glycosylated haemoglobin (in the 12–14 years group), higher values for DBP (15–16 years group), and girls having higher tricipital skinfold measurement in these ages, these results suggest the presence of gender differences in development from preadolescence to adolescence. The difference in parameter values could be due to the difference in growth between boys and girls.

The peak prevalence of overweight in girls tends to occur almost 2 years earlier than in boys (10 vs. 12 years), coinciding with the age of onset of pubertal development, one of the critical moments of body fat gain [48]. Gender differences in body composition are mainly attributed to the action of gender steroid hormones, which drive differences during pubertal development [49]. However, the abdominal circumference patterns differ from what other studies show, with adolescent females gaining considerable amounts of fat but relatively little lean while males show the opposite pattern [50,51]. This may be because the percentage of boys who are overweight/obese is higher than the percentage of girls who are overweight/obese.

Abnormal fat gains during puberty may also reflect an increased risk of developing cardiovascular disease [52].

One of the major limitations of this study was the lack of consideration of diet and eating habits or physical activity levels, which are key factors in glycaemic control.

The main strength of the study lies in the representativeness of the sample by gender and age (4130 adolescents aged 12–17), which allows for the provision of relevant data. However, this study has several limitations. The study lacks data for children under the age of 12, so it would be interesting to study this trend at the youngest age. There is a lack of equity in the percentages of obese/overweight and normal-weight boys and girls. In addition, follow-up data are not available, so its cross-sectional design does not allow for establishing causality, and given that this study population covered a well-characterised cohort of children from Extremadura, that may limit the generalisability of the outcomes to other populations.

Therefore, future studies focusing on the monitoring of HbA1c, blood pressure, triceps skinfold, and abdominal circumference could be studied to determine the existence of causality between the data and age and gender. In addition, it would be interesting to include children’s ages in future studies to know the evolution of these parameters from childhood to adolescence.

## 5. Conclusions

Considering our findings, we conclude that adolescent boys have greater SBP, HbA1c, and waist circumference than girls. However, adolescent girls present a higher triceps skinfold. Furthermore, overweight/obese adolescents show higher SBP, DBP, tricipital skinfold, and abdominal circumference compared to their normal-weight counterparts in both gender, except for DBP in boys aged 15–17 years. Moreover, the positive relationship between blood pressure, tricipital skinfold, and abdominal circumference in overweight/obese children may highlight the need to use anthropometric measures to assess body fat distribution and cardiovascular risk to develop effective strategies to reduce cardiovascular risk in this population.

## Figures and Tables

**Table 1:** Descriptive statistics and between-gender comparison for HbA1c, SBP, DBP, triceps skinfold, and abdominal circumference considering the total sample.

Gender	Male (*n* = 2110)	Female (*n* = 1983)		
12–14 years	Mean (SD)	Mean (SD)	*p*-value	*Hedge’s g*
N	1149	1081		
HbA1c (%)	5.11 (0.32)	5.03 (0.28)	<0.001	0.27 (0.18 to 0.35)
SBP (mmHg)	105.3 (13.3)	102.6 (12.3)	<0.001	0.21 (0.13 to 0.29)
DBP (mmHg)	59.1 (9.1)	58.7 (8.5)	0.272	0.05 (−0.03 to 0.13)
Triceps skinfold (mm)	16.4 (8.7)	18.1 (7.3)	<0.001	−0.21 (−0.29 to −0.13)
Abdominal circumference (cm)	71.0 (10.5)	69.0 (9.9)	<0.001	0.19 (0.11 to 0.28)
15–17 years	Mean (SD)	Mean (SD)	*p*-value	*Hedge’s g*
N	961	902		
HbA1c (%)	5.09 (0.26)	5.00 (0.34)	<0.001	0.30 (0.21 to 0.39)
SBP (mmHg)	111.7 (12.8)	103.4 (12.2)	<0.001	0.66 (0.57 to 0.76)
DBP (mmHg)	62.9 (9.1)	59.1 (8.8)	<0.001	0.42 (0.33 to 0.52)
Triceps skinfold (mm)	16.1 (8.0)	19.0 (7.2)	<0.001	−0.38 (−0.48 to −0.29)
Abdominal circumference (cm)	75.2 (9.8)	71.2 (10.1)	<0.001	0.40 (0.30 to 0.49)

DBP, diastolic blood pressure; HbA1c, glycosylated haemoglobin; SBP, systolic blood pressure.

**Table 2:** Descriptive statistics and between-bodyweight status comparison for HbA1c, SBP, DBP, triceps skinfold, and abdominal circumference stratified by age group in males.

	Overweight/Obese	Normal-Weight		
N (%)	759 (35.97)	1351 (64.03)		
12–14 years	Mean (SD)	Mean (SD)	*p*-value	*Hedge’s g*
N	461	688		
HbA1c (%)	5.11 (0.27)	5.11 (0.35)	0.397	0.00 (−0.12 to 0.12)
SBP (mmHg)	108.5 (13.6)	103.1 (12.7)	<0.001	0.41 (0.29 to 0.53)
DBP (mmHg)	60.7 (9.4)	58.1 (8.7)	<0.001	0.29 (0.17 to 0.41)
Triceps skinfold (mm)	22.9 (8.3)	12.0 (5.7)	<0.001	1.58 (1.44 to 1.71)
Abdominal circumference (cm)	79.1 (10.8)	65.6 (5.8)	<0.001	1.65 (1.52 to 1.79)
15–17 years	Mean (SD)	Mean (SD)	*p*-value	
N	298	663		
HbA1c (%)	5.10 (0.26)	5.09 (0.26)	0.459	0.04 (−0.10 to 0.18)
SBP (mmHg)	117.1 (12.6)	109.3 (12.2)	<0.001	0.63 (0.49 to 0.77)
DBP (mmHg)	65.3 (9.3)	61.8 (8.9)	0.001	0.38 (0.25 to 0.52)
Triceps skinfold (mm)	21.5 (8.0)	12.2 (6.1)	<0.001	1.39 (1.24 to 1.54)
Abdominal circumference (cm)	84.8 (9.6)	70.8 (6.1)	<0.001	1.90 (1.74 to 2.06)

DBP, diastolic blood pressure; HbA1c, glycosylated haemoglobin; SBP, systolic blood pressure.

**Table 3:** Descriptive statistics and between-bodyweight status comparison for HbA1c, SBP, DBP, triceps skinfold, and abdominal circumference stratified by age group in females.

	Overweight/Obese	Normal-Weight		
N (%)	551 (25.97)	1468 (74.04)		
12–14 years	Mean (SD)	Mean (SD)	*p*-value	*Hedge’s g*
N	326	755		
HbA1c (%)	5.04 (0.27)	5.03 (0.28)	0.563	0.04 (−0.09 to 0.17)
SBP (mmHg)	105.3 (13.0)	101.4 (11.8)	<0.001	0.32 (0.19 to 0.45)
DBP (mmHg)	60.1 (8.9)	58.0 (8.2)	<0.001	0.24 (0.11 to 0.37)
Triceps skinfold (mm)	24.4 (6.6)	15.4 (5.7)	<0.001	1.50 (1.35 to 1.64)
Abdominal circumference (cm)	78.2 (9.7)	65.1 (6.9)	<0.001	1.66 (1.51 to 1.81)
15–17 years	Mean (SD)	Mean (SD)	*p*-value	
N	189	713		
HbA1c (%)	5.03 (0.42)	5.0 (0.32)	0.429	0.09 (−0.07 to 0.25)
SBP (mmHg)	106.7 (13.0)	102.6 (11.9)	<0.001	0.34 (0.18 to 0.50)
DBP (mmHg)	60.6 (9.3)	58.7 (8.6)	0.009	0.21 (0.05 to 0.37
Triceps skinfold (mm)	26.0 (7.2)	17.1 (6.0)	<0.001	1.42 (1.25 to 1.59)
Abdominal circumference (cm)	81.2 (10.4)	68.6 (8.1)	<0.001	1.34 (1.17 to 1.51)

DBP, diastolic blood pressure; HbA1c, glycosylated haemoglobin; SBP, systolic blood pressure.

## Data Availability

The datasets used during the current study are available from the corresponding author upon reasonable request.
